# 3D Cocultures of Osteoblasts and *Staphylococcus aureus* on Biomimetic Bone Scaffolds as a Tool to Investigate the Host–Pathogen Interface in Osteomyelitis

**DOI:** 10.3390/pathogens10070837

**Published:** 2021-07-03

**Authors:** Raffaella Parente, Valentina Possetti, Maria Lucia Schiavone, Elisabetta Campodoni, Ciro Menale, Mattia Loppini, Andrea Doni, Barbara Bottazzi, Alberto Mantovani, Monica Sandri, Anna Tampieri, Cristina Sobacchi, Antonio Inforzato

**Affiliations:** 1 IRCCS Humanitas Research Hospital, 20089 Rozzano, Italy; Raffaella.Parente@humanitasresearch.it (R.P.); valentina.possetti@humanitasresearch.it (V.P.); marialucia.schiavone@humanitasresearch.it (M.L.S.); mattia.loppini@hunimed.eu (M.L.); andrea.doni@humanitasresearch.it (A.D.); barbara.bottazzi@humanitasresearch.it (B.B.); alberto.mantovani@humanitasresearch.it (A.M.); 2 National Research Council-Institute for Genetic and Biomedical Research (CNR-IRGB), Milan Unit, 20089 Rozzano, Italy; ciro.menale@unina.it; 3 National Research Council-Institute of Science and Technology for Ceramics (CNR-ISTEC), 48018 Faenza, Italy; elisabetta.campodoni@istec.cnr.it (E.C.); monica.sandri@istec.cnr.it (M.S.); anna.tampieri@istec.cnr.it (A.T.); 4 Department of Clinical Medicine and Surgery, University of Naples “Federico II”, 80131 Naples, Italy; 5 Department of Biomedical Sciences, Humanitas University, 20072 Pieve Emanuele, Italy; 6 The William Harvey Research Institute, Queen Mary University of London, London E1 4NS, UK; 7 National Research Council-Institute of Nanostructured Material (CNR-ISMN), 40129 Bologna, Italy

**Keywords:** *Staphylococcus aureus*, osteomyelitis, osteoblast-like cells, host–pathogen interface, 3D models, biomimetic bone scaffolds

## Abstract

Osteomyelitis (OM) is an infectious disease of the bone primarily caused by the opportunistic pathogen *Staphylococcus aureus* (SA). This Gram-positive bacterium has evolved a number of strategies to evade the immune response and subvert bone homeostasis, yet the underlying mechanisms remain poorly understood. OM has been modeled in vitro to challenge pathogenetic hypotheses in controlled conditions, thus providing guidance and support to animal experimentation. In this regard, traditional 2D models of OM inherently lack the spatial complexity of bone architecture. Three-dimensional models of the disease overcome this limitation; however, they poorly reproduce composition and texture of the natural bone. Here, we developed a new 3D model of OM based on cocultures of SA and murine osteoblastic MC3T3-E1 cells on magnesium-doped hydroxyapatite/collagen I (MgHA/Col) scaffolds that closely recapitulate the bone extracellular matrix. In this model, matrix-dependent effects were observed in proliferation, gene transcription, protein expression, and cell–matrix interactions both of the osteoblastic cell line and of bacterium. Additionally, these had distinct metabolic and gene expression profiles, compared to conventional 2D settings, when grown on MgHA/Col scaffolds in separate monocultures. Our study points to MgHA/Col scaffolds as biocompatible and bioactive matrices and provides a novel and close-to-physiology tool to address the pathogenetic mechanisms of OM at the host–pathogen interface.

## 1. Introduction

Osteomyelitis (OM) is an inflammatory disease of the bone caused by a wide range of opportunistic pathogens, including the Gram-positive bacteria *Staphylococcus aureus* (SA), *S. epidermidis*, *Streptococcus pyogenes*, and *Pseudomonas aeruginosa* and the Gram-negative *Escherichia coli* [1]. In children OM primarily results from the hematogenous spread of the pathogen from distant sites of infection [2], whereby in adults most cases of OM originate from bone contamination during invasive surgical procedures, particularly, fracture fixation and arthroplasty [3]. Regardless of how the infectious agent colonizes the bone, a series of pathogenetic events follows that eventually leads to dramatic alterations and a loss of diverse bone compartments, including trabecular and cortical bone, marrow, periosteum, and the surrounding soft tissue [4]. Aggressive antimicrobial treatments are the standard of care for OM [5], however antibiotic resistant strains are emerging that undermine efficacy of the currently available therapeutic options [6].

The ubiquitous commensal SA is responsible for up to 75% of OM cases [7], likely due to a remarkable tropism for bone tissue and the exploitation of several strategies of adaptation and resistance [8,9,10,11]. The bone-invasive properties of SA likely arise from a variety of microbial surface components recognizing adhesive matrix molecules (MSCRAMMs) that mediate the recognition of and adherence to bone extracellular matrix (BEM) components, like type I collagen, bone sialoprotein, osteopontin, fibronectin, and laminin [12]. Following adherence to the BEM, SA can target, for infection, a number of resident cell types, including bone forming (osteoblasts, OBs) and resorbing (osteoclasts, OCs) cells, where OB-dependent responses are believed to play a major role in OM pathogenesis [13]. Indeed, recognition of pathogen-associated molecular patterns (PAMPs) by OB-borne and OB-secreted pattern recognition molecules (PRMs) prompts synthesis and release of chemokines, cytokines, and growth factors (e.g., TNF-α and TGF-β) [14] that, in turn, promote recruitment and activation of immune cells (particularly, macrophages and neutrophils) [15,16]. Among the range of molecules expressed by OB lineage cells besides immune ones, the soluble PRM pentraxin 3 (PTX3) has been recently proposed as a specific and independent diagnostic and prognostic biomarker in opportunistic infections [17], including those sustained by SA [18,19].

Furthermore, SA decreases both the activity and viability of OBs, via apoptosis-dependent [20,21] and apoptosis-independent [22,23] pathways, and alters redox homeostasis in these cells [24]. In addition, following an infection with SA, OBs produce more receptor activator of NF-κB ligand (RANKL) and less osteoprotegerin (OPG), which, in turn, promotes osteoclastogenesis and bone resorption [25,26]. These processes collectively contribute to perturbation and even loss of bone homeostasis [27], and highlight the key roles played by OBs at the host–pathogen interface in OM pathogenesis [13]. It is therefore conceivable that research into the crosstalk between OBs and SA will deliver novel information on the molecular mechanisms of OM. In this regard, animal modeling of the disease are invaluable as they integrate the structural and functional complexity of the natural bone, nonetheless addressing the OB–SA interface in vivo might be problematic due to the confounding and redundancy effects contributed by other cells of the bone [28]. On the other hand, conventional bidimensional (2D) in vitro models, based on either primary or immortalized cells (such as the human MG63 and murine MC3T3-E1 cell lines) allow direct investigation of the host–pathogen crosstalk (including formation of biofilms [3] and adaptation to slow-growing small colony variants, SCVs [29,30]) in a controlled setting. However, they are far from reproducing the physical and chemical cues of the skeletal tissue, with major regard to the organization of OBs in the bone matrix [3,31,32]. More recently, three-dimensional (3D) models of OM have been proposed that recapitulate some aspects of the bone tissue architecture and cellular complexity. A common limitation of the currently available 3D models of OM is poor reproduction, in the applied in vitro settings, of the natural BEM, which is an essential player in SA adhesion, biofilm formation, and infection of bone cells [12].

To overcome this issue, here we exploited biomimetic hybrid composite scaffolds manufactured with a biomineralization process that involves direct nucleation of Mg-doped hydroxyapatite/collagen I (MgHA/Col) [21]. These scaffolds incorporate the major organic (collagen I) and mineral (hydroxyapatite) components of the authentic bone matrix in a porous structure, which likely provides not only structural support but also biomechanical and chemical clues to the cultured cells, different to other synthetic matrices that lack either hydroxyapatite or collagen or both [6,33]. Taking advantage of these unique features of the MgHA/Col scaffolds, here we implemented a novel 3D model of OM based on murine osteoblastic MC3T3-E1 cells infected with a clinical isolate of SA. Functional adaptation of cells to the applied 3D environment was investigated, which highlighted major differences to conventional 2D cultures on untreated plastic surfaces. Additionally, early phases of the SA infection were studied that suggest matrix-dependent effects on the expression of primary mediators of the OB function. In addition, we found profound differences in the expression of SA virulence genes known to participate in adhesion to the bone matrix, recognition of OBs, and biofilm formation. Therefore, we established a novel strategy to model OM in vitro that is likely to provide pathologically relevant insights into molecular and cellular processes of OM at the host–pathogen interface.

## 2. Results

### 2.1. Adaptation of MC3T3 Cells to 3D Cultures on MgHA/Col Scaffolds

**Figure 1:**
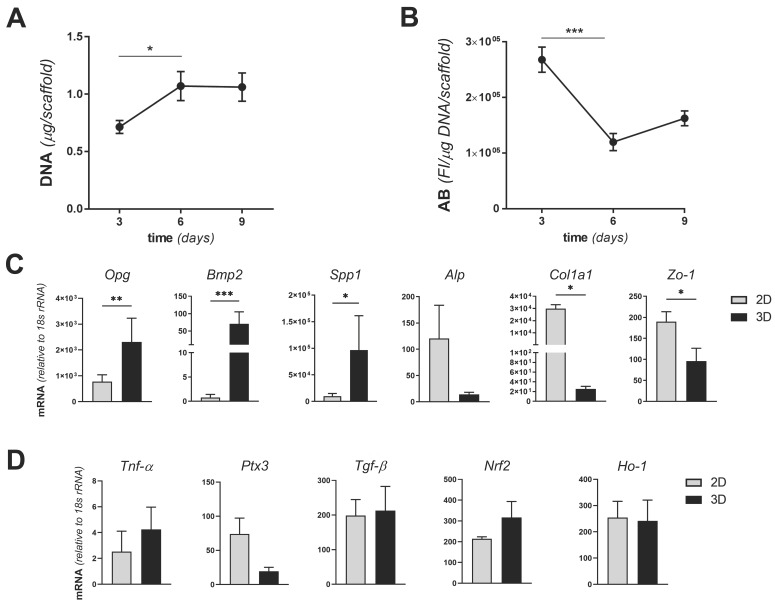
Proliferation, metabolic activity, and gene expression of MC3T3-E1 cell cultures on MgHA/Col 3D scaffolds. MC3T3-E1 cells were seeded on MgHA/Col 3D scaffolds at a concentration of 1 × 10^6^ cells/scaffold and cultured for 9 days. (**A**) Cells were lysed and genomic DNA was quantitated using the PicoGreen assay at the indicated time points. Results are expressed as micrograms of DNA/scaffold. (**B**) The metabolic activity of viable cells was measured at 3, 6, and 9 days from seeding using the Alamar Blue (**A**,**B**) assay. Results are expressed as fluorescence intensity (FI in arbitrary units)/μg of DNA/scaffold. Data in A and B are from 2 independent experiments performed in duplicate, *n* = 4, mean ± SEM. (**C**,**D**) Total RNA was extracted from MC3T3-E1 cells at day 6 from seeding (i.e., in stationary growth phase) and transcription (mRNA) of the indicated osteogenic (osteoprotegerin, *Opg*; bone morphogenetic protein 2, *Bmp2*; secreted phosphoprotein 1, *Spp1*; alkaline phosphatase, *Alp*; collagen type 1 α1 chain, *Col1a1*) and adhesion (zonula occludens-1, *Zo-1*) (**C**) and inflammatory (tumor necrosis factor-α, *Tnf-α*; pentraxin 3, *Ptx3*; transforming growth factor-β, *Tgf-β*) and oxidative stress response (nuclear factor erythroid 2–related factor 2, *Nrf2*; heme oxygenase 1, *Ho-1*) (**D**) genes (mRNA levels) was determined by qRT-PCR. Results are expressed as normalized values based on 18S rRNA (3 independent experiments performed in duplicate, *n* = 6, mean± SEM). In panels A to C, *** *p* < 0.001, ** *p* < 0.01, and * *p* < 0.05, Mann–Whitney test.

To establish an MgHA/Col-based 3D model of bone infection, the number and metabolic activity of MC3T3-E1 cells were assessed when these were cultured on biomimetic scaffolds in the absence of SA. A total of 1 × 10^6^ MC3T3-E1 cells/scaffold were seeded and genomic DNA (as an index of cell proliferation) was quantitated at 3, 6, and 9 days from seeding using the PicoGreen assay. Cells proliferated from day 3 to 6 and no further expansion was observed after day 6 (up to day 9), when a stationary growth phase was achieved (Figure 1A). As opposed to this, the rate of reduction of resazurin to resorufin (that generates a fluorescence signal in the applied Alamar Blue assay and is an index of metabolic activity) decreased from day 3 to 6 and remained stable afterwards (up to day 9) (Figure 1B). These findings indicate that the MC3T3-E1 cell line expands on (Figure 1A) and metabolically adapts to (Figure 1B) the MgHA/Col scaffolds, which therefore proved to be biocompatible in the applied experimental conditions. Prompted by these observations, we analyzed the expression of major osteogenic, inflammatory and oxidative stress response-related genes in MC3T3-E1 cells cultured in stationary conditions on MgHA/Col scaffolds (i.e., at 6 days from seeding), and compared it with that of the same cells in conventional subconfluent 2D cultures (with comparable cell counts). We found significantly higher mRNA levels of osteoprotegerin (*Opg*, which together with *Rankl* is a key mediator of the OB–OC crosstalk and bone homeostasis [34]), bone morphogenetic protein 2 (*Bmp2*, which plays an important role in inducing the osteogenic differentiation of mesenchymal stem cells [35]), and secreted phosphoprotein 1 (*Spp1*, coding for osteopontin, a marker of late osteogenic differentiation involved also in infection [26,36]), in 3D- versus 2D-cultured cells. This result was in agreement with the previously reported osteoinductive properties of MgHA/Col scaffolds [37,38,39]. Additionally, we observed lower expression both of the alkaline phosphatase (*Alp*) and collagen type 1 α1 chain (*Col1a1*) genes when cells were adapted to the 3D setting, as compared to cultures on plastic surfaces (Figure 1C). These genes are markers of the preosteoblast and mature osteoblast phase. In particular, *Alp* is known to be reduced in mature as compared to immature osteoblasts [38], it is plausible that the MgHA/Col scaffolds favor transition of the MC3T3-E1 cells towards a more advanced stage of differentiation, possibly via BEM–cell interactions. This might also explain why *Col1a1* was downregulated when cells were grown in the collagen-rich microenvironment of the scaffolds (Figure 1C). In line with this interpretation, expression of zonula occludens-1 (*Zo-1*, involved in cell–cell interaction) was reduced in our 3D setting, suggesting that the MC3T3-E1 cells are engaged in BEM–cell rather than cell–cell contacts when they are seeded on MgHA/Col matrices (Figure 1C). As opposed to this, no differences were found between 3D and 2D cultures in the expression of selected inflammatory markers, including tumor necrosis factor-α (*Tnf-α*), pentraxin 3 (*Ptx3*), and transforming growth factor-β (*Tgf-β*). In addition, we measured the expression levels of genes acting in a master regulator pathway of the redox status of the cells, modulated also by SA infection of bone cells, i.e., the Keap1-Nrf2 pathway [40]. In our experimental conditions, the nuclear factor erythroid 2–related factor 2 (*Nrf2*) and heme oxygenase 1 (*Ho-1*) gene expression level was unchanged in the two culture models (Figure 1D). Overall, these data indicate that the MgHA/Col matrices not only are compatible with 3D culturing of MC3T3-E1 cells, but also provide physical and chemical cues that reproduce some aspects of the BEM–cell interface in the natural bone.

### 2.2. SA Growth on MgHA/Col Scaffolds

**Figure 2:**
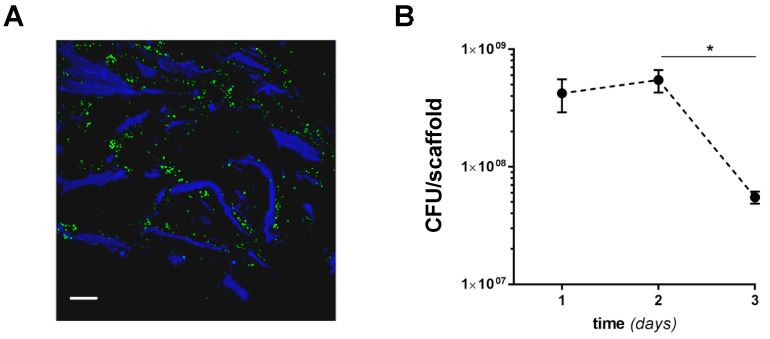
SA colonization of MgHA/Col scaffolds. MgHA/Col empty scaffolds were inoculated with 1.6 × 10^8^ bacteria/scaffold, and SA colonization was assessed using fluorescence microscopy and CFU counting. (**A**) Following fixation and sectioning, SA cells on scaffolds were revealed using a FITC-conjugated anti-*Staphylococcus aureus* antibody (green) and collagen was recorded as blue autofluorescence. An image of SA-inoculated scaffolds harvested at 24 h from inoculum is shown that is representative of at least 3 independent experiments (scale bar: 64 µm). (**B**) CFUs were measured at 1, 2, and 3 days from inoculum. Results are expressed as CFU/scaffold (3 independent experiments performed in duplicate or triplicate, *n* = 6–8, mean ± SEM). * *p* < 0.05, one-way ANOVA test with Dunn’s multiple comparisons test.

We next investigated the adaptation of SA to the MgHA/Col matrices by assessing bacterial growth and scaffold colonization for 3 days from inoculum (a time window that spans the early phases of OM pathogenesis in vivo, when bacteria adhere to the natural bone [41]). To this end, 160 × 10^6^ bacteria were seeded on empty scaffolds, and SA colonization of the bone-like matrix in the absence of cells was assessed by means of fluorescence microscopy and colony forming unit (CFU) counting. Widespread diffusion of bacteria along the scaffold’s collagen fibers was apparent in immunofluorescence images taken 24 h postinfection (Figure 2A) and at later time points (data not shown). The number of SA CFUs retrieved from the MgHA/Col scaffolds was stable within 2 days from inoculum, indicating that the biomimetic matrix did not affect SA viability in this timeframe (Figure 2B). However, at day 3 the CFU count decreased, possibly due to partial biodegradation of and magnesium release by the MgHA/Col scaffolds (Figure 2B) [33].

### 2.3. The 2D and 3D Cocultures of MC3T3-E1 Cells and SA

**Figure 3:**
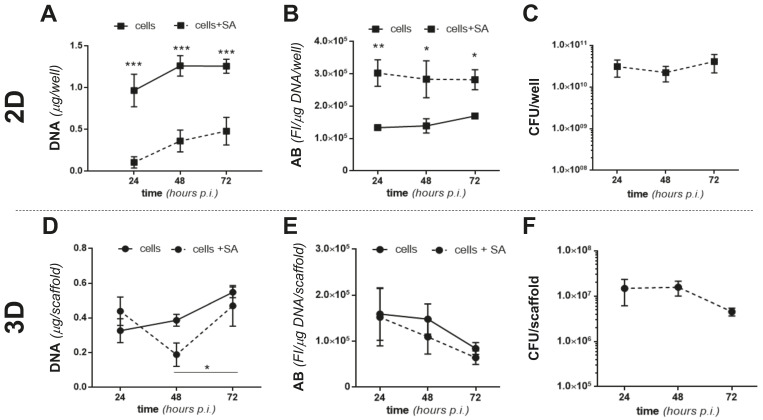
Proliferation and metabolic activity of MC3T3-E1 and SA in 2D and 3D cocultures. MC3T3-E1 cells were seeded in 48 well plates (2D) or on MgHA/Col 3D scaffolds (3D) and cultured for 1 or 6 days, respectively, to achieve comparable cell counts (3 × 10^5^ cells/well or scaffold). Either live or inactivated (fixed) SA was then inoculated (MOI of 160:1) and cocultured with MC3T3-E1 cells for 72 h. (**A**,**D**) DNA from MC3T3-E1 cells challenged with live SA was quantitated using the PicoGreen assay at the indicated time points (cell proliferation). Results are expressed as micrograms of DNA/well or scaffold in the absence (solid line) or presence (dotted line) of SA in the 2D (**A**) and 3D (**D**) settings. (**B**,**E**) The metabolic activity of MC3T3-E1 cells challenged with inactivated SA was measured at the indicated time points using the AB assay. Results are expressed as fluorescence intensity (FI in arbitrary units)/μg of DNA/well or scaffold in the absence (full line) or presence (dotted line) of SA in 2D (**B**) and 3D (**E**) experiments. (**C**,**F**) CFU counts from live SA-MC3T3-E1 cocultures at 24, 48, and 72 h from infection in 2D (**C**) or 3D (**F**) settings. In all panels, results are from 2 independent experiments performed in duplicate, triplicate, or quadruplicate, *n* = 4–8, mean ± SEM. *** *p* < 0.001, ** *p* < 0.01, and * *p* < 0.05, two-way ANOVA with Sidak’s multiple comparisons tests.

We then infected MC3T3 cells with SA in 2D and 3D culture conditions and investigated both osteoblast’s responses to the presence of SA and bacterial survival in the presence of MC3T3-E1 cells. Briefly, MC3T3-E1 cells were cultured for 24 h in 48 well plates (2D) or 6 days on MgHA/Col scaffolds (3D, to achieve a stationary growth phase; see Figure 1A), then challenged with SA at a multiplicity of infection (MOI) of 160:1. In the 2D setting, SA significantly reduced proliferation of the osteoblastic cells (Figure 3A) and increased their metabolic activity (Figure 3B) at 24, 48, and 72 h from infection. Additionally, the number of viable bacteria did not change in this time window in the 2D experiments (Figure 3C). As opposed to this, in the 3D model SA caused a reduction in MC3T3-E1 proliferation at 48 h from infection only, while 24 h later (i.e., 72 h from infection) the osteoblastic cells recovered (Figure 3D), likely due to poor SA adhesion and growth in these conditions (see below). Furthermore, the metabolic activity of the MC3T3-E1 cells was not affected by SA (Figure 3E). In addition, in this setting, the CFU count was unchanged throughout the coculture (Figure 3F) and less bacteria adhered to the MgHA/Col scaffolds in the presence of the MC3T3-E1 cells (see the 24 h time point in Figure 3F and Figure 2B for a comparison), suggesting that preincubation of the scaffolds with these cells might impair adhesion of SA to the 3D matrix. Moreover, our findings point to this osteoblastic cell line manifesting different behaviors in terms of survival and metabolic activity in 2D and 3D settings both in the presence and in absence of SA, further highlighting the biological relevance of the BEM–cell interface, which is missing in the 2D cocultures.

### 2.4. Colonization of MgHA/Col Scaffolds by MC3T3 and SA

**Figure 4:**
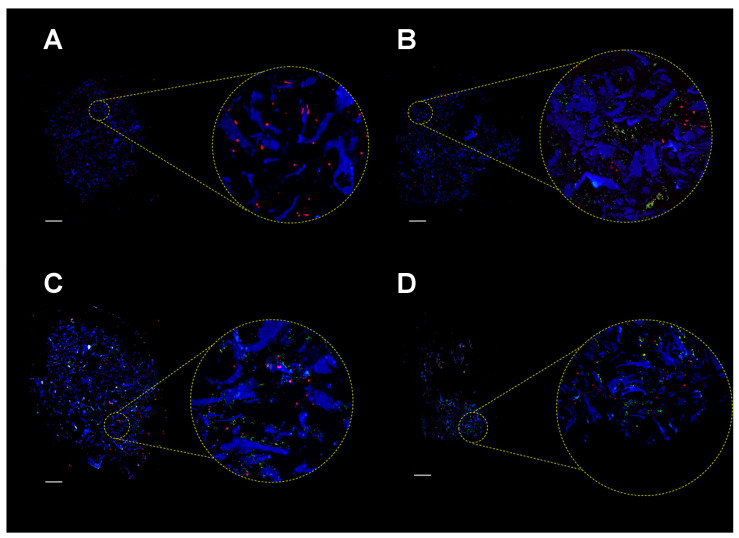
Immunofluorescence analysis of MgHA/Col scaffolds seeded with MC3T3-E1 cells and SA bacteria. MC3T3-E1 cells were seeded on MgHA/Col 3D scaffolds and cultured for 6 days. Live SA was then inoculated (MOI of 160:1), and cocultured with MC3T3-E1 cells for 72 h. Fluorescence microscopy images (representative of 3 independent experiments) are shown that were obtained from scaffolds seeded with MC3T3-E1 cells only (**A**) or with MC3T3 and SA after 24 (**B**), 48 (**C**), and 72 (**D**) hours of coculture. Bacteria were revealed using a FITC-conjugated anti-*Staphylococcus aureus* antibody (green), nuclei were stained with propidium iodide (red), and collagen autofluorescence was collected as a blue signal. Yellow spots in the images are due to colocalization of green and red fluorescence, and likely indicate SA cells. Scale bar represents 400 µm.

To assess colonization of the MgHA/Col scaffolds by MC3T3-E1 cells and SA, we analyzed cell-seeded matrices before infection and at different time points from SA inoculation, using fluorescence microscopy and scanning electron microscopy (SEM). Immunofluorescence analysis of scaffolds’ sections showed that MC3T3-E1 cells adhered to the MgHA/Col meshwork and localized on the collagen fibers (Figure 4A). Additionally, upon infection, bacteria spread throughout the scaffold, localizing both on the collagen matrix and near MC3T3-E1 cells, and formed colonies (Figure 4B–D).

**Figure 5:**
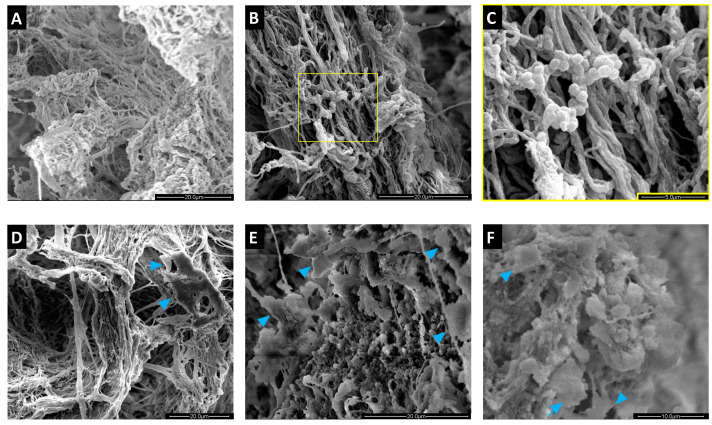
Scanning electron microscopy (SEM) analysis of MgHA/Col scaffolds seeded with MC3T3-E1 cells and SA bacteria. MC3T3-E1 cells were seeded on MgHA/Col 3D scaffolds and cultured for 6 days. Live SA was then inoculated (MOI of 160:1) and cocultured with MC3T3-E1 cells for 72 h. SEM images (representative of 3 independent experiments) are reported that show: (**A**) the peculiar microarchitecture of an empty MgHA/Col scaffold (scale bar: 20 µm); (**B**,**C**) a scaffold colonized by SA (scale bars: 20 µm and 5 µm, respectively); (**D**) a scaffold seeded with MC3T3-E1 cells only (scale bar: 20 µm); (**E**,**F**) SA infection of an MC3T3-E1-seeded scaffold at 24 (**E**) and 72 (**F**) hours post infection (scale bars: 20 µm and 10 µm, respectively). In D, E, and F the blue arrowheads indicate MC3T3-E1 cells.

SEM analysis of empty scaffolds clearly showed the porous structure of these matrices and the spatial organization of the collagen fibers (Figure 5). In this peculiar microenvironment, SA localized on the matrix, maintaining its typical morphology while forming colonies (Figure 5B,C). MC3T3-E1 cells arranged along the collagen fibers (Figure 5D). After infection, close contacts between MC3T3-E1 cells, SA, and the matrix were observed, which were retained throughout the coculture (Figure 5E,F).

### 2.5. Gene Expression and Protein Levels of SA-Infected MC3T3-E1 Cells

**Figure 6:**
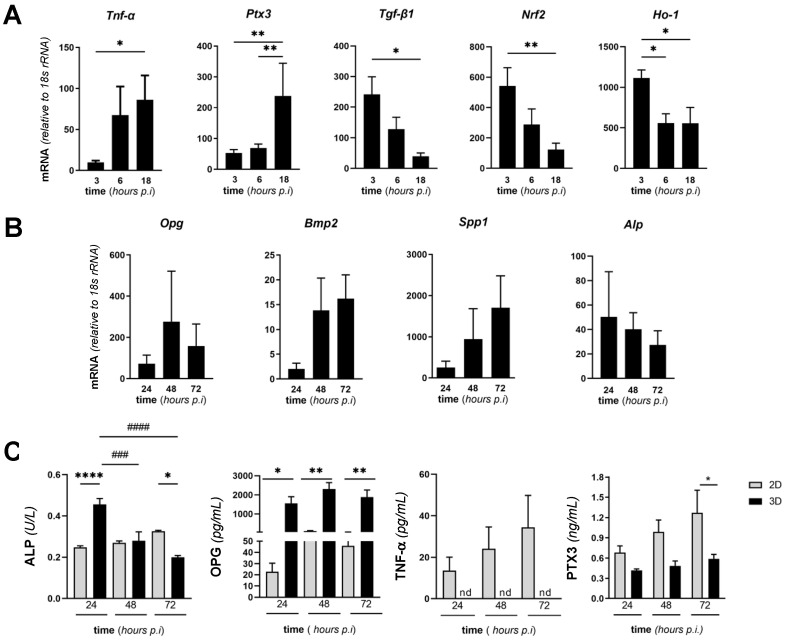
Expression of selected genes and proteins in MgHA/Col-seeded MC3T3-E1 cells infected with SA. MC3T3-E1 cells were seeded on MgHA/Col 3D scaffolds and cultured for 6 days. Live SA was then inoculated (MOI of 160:1) and cocultured with MC3T3-E1 cells up to 72 h. (**A**,**B**) Cells were harvested at the reported time points, total RNA was extracted, and mRNA levels of the indicated genes were measured by qRT-PCR. Data were normalized based on the levels of 18S rRNA, and expressed as mean ± SEM (*n* = 4–6 from 2 to 3 independent experiments performed in duplicate). (**C**) Activity of ALP and concentration of OPG and PTX3 in the conditioned medium of MC3T3-E1/SA cocultures were determined using commercial enzymatic and ELISA kits. Data are from 3 to 4 independent experiments performed in duplicate or triplicate (*n* = 6–8, mean ± SEM). In panels A and B, ** *p* < 0.01 and * *p* < 0.05, one-way ANOVA test with Dunn’s multiple comparisons test. In panel C, **** *p* < 0.0001, *** *p* < 0.001, ** *p* < 0.01, and * *p* < 0.05 (comparison of the observations recorded from 3D and 2D settings at the same time points), and #### *p* < 0.0001, ### *p* < 0.001, ## *p* < 0.01, and # *p* < 0.05 (comparison of the observations recorded at different time points from the same setting, i.e., either 2D or 3D) two-way ANOVA with Sidak’s multiple comparisons test.

To characterize further our in vitro model of SA infection of the bone, we assessed the expression of selected genes in SA-infected MC3T3-E1 cells cultured in 3D conditions at early times (from 3 to 18 h) from infection. In particular, we focused on a set of genes that are paradigms of the cell inflammatory and oxidative stress response. Amongst these, mRNA of the inflammatory cytokine TNF-α increased over time with a peak at 18 h from infection. Accordingly, transcription of *Ptx3*, which is involved in bone remodeling [18,42] and is known to be induced by TNF-α [43] was significantly increased 18 h postinfection. Interestingly, *Tgf-β* mRNA had an opposite trend with a clear reduction at 18 h (Figure 6A). A similar (to *Tgf-β*) expression kinetic was observed for *Nrf2* and its downstream effector *Ho-1*: the former had significantly decreased transcription at 18 h from infection, and the latter was less expressed at 6 h already and had consistently reduced mRNA levels up to 18 h from infection. Comparable results were obtained in the 2D setting (see Figure S2 in Supplementary Materials), with the noticeable exception of *Ptx3*, whose transcription was reduced in these conditions. It is worth noting here that SA-infected MC3T3-E1 cells were less viable in the 2D than in the 3D setting at 24 h from infection (Figure 3A), which made it problematic to perform comparative gene expression analysis at longer times. Prompted by our observation that the MgHA/Col scaffolds affected expression of key osteogenesis-related genes in MC3T3-E1 cells cultured in the absence of SA (see Figure 1C), we extended our investigations to 3D cocultures of MC3T3-E1 and SA. Despite these matrix-dependent effects, the mRNA levels of *Opg*, *Bmp2*, and *Spp1* did not change significantly during the infection (Figure 6B), consistent with the reported antiosteogenic effects of inflammation and infection [44,45]. Additionally, a decremental trend was observed in the transcription of *Alp* (Figure 6B).

Furthermore, we quantitated activity and/or concentration of osteogenic and inflammatory soluble markers in the conditioned medium of SA/MC3T3-E1 cocultures on MgHA/Col scaffolds at 24, 48, and 72 h from bacterial inoculation. Specifically, we determined ALP enzymatic activity (a specific index of the osteogenic activity of MC3T3-E1 cells) and found it to decrease during infection (Figure 6C), consistent with the trend observed at the mRNA level (Figure 6B). Concentration of OPG (a well-characterized inhibitor of osteoclastogenesis and bone resorption) did not change at the observation times (Figure 6C). Similarly, the PTX3 protein was secreted and dosed in the conditioned medium and had an incremental yet not significant trend in the course of the infection (Figure 6C), thus reflecting the gene transcription profile reported in Figure 6B. No changes over time were observed in the concentration/activity of ALP, OPG, PTX3, and TNF-α (which was undetectable in the 3D setting) in the supernatant of 2D cultures (Figure 6C). Furthermore, ALP activity was higher in 3D than 2D conditioned media at 24 h, which was reversed at 72 h, while the concentration of OPG was consistently more elevated in 3D than 2D conditioned media at all time points, and PTX3 was significantly more abundant in 2D than 3D cocultures at 72 h only. These data collectively suggest MgHA/Col-dependent effects on the ability of SA-infected MC3T3-E1 cells to synthesize and release both osteogenic (ALP and OPG) and inflammatory (TNF-α and PTX3) mediators.

### 2.6. Expression of Virulence SA Genes in MC3T3-E1/SA Cocultures

**Figure 7:**
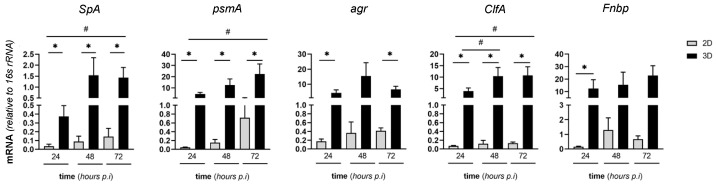
Expression of virulence SA genes in MC3T3-E1/SA cocultures. MC3T3-E1 cells were seeded on MgHA/Col 3D scaffolds, cultured, and challenged with SA as described in Figure 6. Cells were harvested at the reported time points, total RNA was extracted, and mRNA levels of the indicated bacterial genes were measured by qRT-PCR. Data were normalized based on the levels of 16S rRNA, and expressed as mean ± SEM (two independent experiments performed in triplicate or quadruplicate, *n* = 7). ** *p* < 0.01 and * *p* < 0.05, one-way ANOVA test with Dunn’s multiple comparisons test (comparison of the observations recorded at different time points from the same setting, i.e., either 2D or 3D). # *p* < 0.05, Mann–Whitney test (comparison of the observations recorded from 3D and 2D settings at the same time points).

We then evaluated the expression of SA genes coding for key virulence factors both in 2D and in 3D MC3T3-E1/SA cocultures. Specifically, we investigated *Staphylococcus protein* A (*SpA*, a major survival and virulence factor of SA), phenol soluble modulin A (*psmA*, a bacterial toxin with cytolytic activity), accessory gene regulator (*agr*, an essential component of the SA quorum-sensing system), clumping factor A (*ClfA*), and fibronectin binding protein (*Fnbp*), which are both involved in bacteria clumping and fibronectin binding [26,41,46,47]. Our data indicate increased transcription of all these genes during infection of the MC3T3-E1 cells, except for *agr* and *Fnbp* whose mRNA levels were unchanged (Figure 7). Of note, all these genes had higher expression in the 3D setting than in 2D cultures (Figure 7) and their transcription did not change in the course of the infection in the 2D model (as opposed to 3D).

## 3. Discussions

*Staphylococcus aureus* is amongst the most common human pathogens and a primary etiologic agent of bone infections, with major regard to OM [27], whereby it is responsible for alterations and loss of the bone cells homeostasis [13]. The pathogenetic mechanisms of this debilitating disease are not fully elucidated and deserve urgent investigations. Traditional in vitro models to study the host–pathogen interphase in OM are based on 2D monolayers of cells. These experimental settings are far from reproducing the structural complexity of the bone microenvironment, which poses severe limitations on the relevance of such modeling strategies to the human pathology [6]. Three-dimensional models of OM have been reported that mimic some characteristics of the BEM. Kavanagh et al. developed a 3D model of bone infection to examine colonization and infection of MC3T3 cells by SA on EDAC (1-ethyl-3-(3-dimethylaminopropyl)carbodiimide) cross-linked glycosaminoglycan collagen scaffolds [6]. In this setting, SA-infected MC3T3-E1 cells had increased bone matrix-deposition activity, a phenomenon that mimics the human pathology where OBs counteract infection-induced bone loss by increasing bone mineralization [6]. To study in vitro postoperative SA biofilm-dependent osteomyelitis, Raic et al. implemented a 3D model based on human hematopoietic stem and progenitor cells (HSPCs) and mesenchymal stromal cells (MSCs) cultured on cationized bovine serum albumin (cBSA) scaffolds, which simulates implant-associated osteomyelitis [48]. Additional strategies have been exploited to model OM in vitro using 3D systems (reviewed in [3,49]), which allowed studying specific pathogenetic mechanisms of the disease, including intracellular colonization of non-professional phagocytes (like OBs, OCs, and osteocytes), a process that is believed to contribute to bacterial persistence, chronic infection, and antibiotic resistance [50,51,52]. However, currently available 3D models of OM share a common limitation, i.e., poor reproduction of the natural BEM, which is an essential player in SA adhesion, biofilm formation, and infection of bone cells [12].

To overcome this limitation, here we propose a composite system in which 3D biomimetic scaffolds made of MgHA/Col mixed matrix are seeded with the murine MC3T3-E1 osteoblast precursor cell line and infected with SA. The MgHA/Col scaffolds have already demonstrated high biocompatibility with different osteogenic cells, such as murine and human mesenchymal stem cells and human osteosarcoma cell lines (MG63 and SAOS-2) [37,38,39,53,54], while to the best of our knowledge the osteoblast cell line MC3T3-E1 derived from primary mouse calvaria has been used here for the first time. In line with previous work on other osteogenic cells, we found that MC3T3-E1 cells adapted to the 3D culture on MgHA/Col scaffolds, proliferated, were metabolically active, and shaped their gene expression according to a more advanced differentiation state by increasing, for instance, the mRNA levels of *Bmp2* and *Spp1* genes, as compared to 2D conditions (Figure 1). Notably, 3D culturing did not activate inflammatory and antioxidant responses, which are expected to inhibit the osteogenic functions of OBs [44,45].

SA-induced orthopedic infections constitute a major health threat; therefore, much effort has been devoted to unravel the underlying pathogenetic mechanisms. A variety of experimental settings have been implemented to mimic the host–pathogen interaction, comprising collagen glycosaminoglycan scaffolds, titanium substrates coated with MgHA or silicon and copper, or polylactic acid nanofibers coated with a silver-releasing polymer [6,55,56]. Nevertheless, also in this respect the use of MgHA/Col scaffolds, displaying the key features of the bone matrix, is a novelty and an important advancement. In fact, although valuable for specific research purposes (e.g., biofilm formation and infection of OB by SA), those systems lack either the mineral or the organic component or the spatial cues encountered by SA in the natural bone. In our MgHA/Col scaffolds, all these features are present and ideally organized due to a bioinspired manufacturing process. By using different techniques, we showed that these scaffolds per se support both survival and colonization of SA (Figure 2 and Figure 5), which allowed the subsequent development of MC3T3-E1/SA 3D cocultures (Figure 4 and Figure 5) and comparison with parallel 2D experiments (Figure 3). This highlighted important differences between the 2D and 3D settings. First, the MC3T3-E1 cells were more sensitive to SA infection when cultured in 2D conditions, as demonstrated by a strong reduction in cell survival, in accordance with previous studies [20,21,22,23,24], and an increase in metabolic activity (Figure 3). Second, we focused on the early phases of the SA infection (that are relevant to bacterial adhesion and biofilm formation [3,41]), and observed different kinetics (between 2D and 3D) for the expression of inflammatory and antioxidant genes (Figure 6), with major regard to the long pentraxin PTX3, a well-established PRM with emerging roles in bone physiology and pathology [18,42]. Regarding the bacterial counterpart in our coculture models, lower bacterial loads were found in the 3D compared to the 2D setting at all time points (Figure 3). Additionally, and more interestingly, SA genes coding for key virulence factor (SpA, PSM-A, agr, ClfA, and FnBP) had much more increased expression in the 3D than in the 2D setting (Figure 7). Possible explanations for these divergent responses could be a different engagement of MC3T3-E1 and SA in cell–cell and cell–matrix interactions in the two models, or a diverse cell–bacteria interplay affecting viability, gene expression, and virulence. Interestingly, a recent paper demonstrated that magnesium-based composite scaffolds had a bacterial inhibition potential proportional to their magnesium content. In fact, sustained ion release over time owing to scaffold dissolution caused alkalinization of the supernatant, generating a poorly permissive milieu for the bacteria [33]. We may hypothesize that a similar situation occurred in our 3D model, particularly in the presence of MC3T3-E1, which may have contributed to scaffold remodeling along the culture.

Previous studies demonstrated that OB infection by SA induces inflammatory cytokine release and alters the osteogenic functions of these cells [8,9,10,11]. These findings were recapitulated in our 3D experimental setting, thus supporting the validity of this model as a new tool to model and investigate the SA–OM. In this regard, the bone biominetic microenvironment of the MgHA/Col scaffolds here described appears closer to the in vivo situation than conventional 2D settings and other 3D models described in the literature and deserves further investigation by means of omics approaches targeted both to the osteoblastic cell and to the bacterial component.

We recognize that our work had some limitations. Specifically, a single bacterial strain was used in our coculture experiments. However, in a recent work, Musso and colleagues [40] infected the human MG63 osteoblast-like cell line with four methicillin-resistant and one methicillin-sensitive SA clones in a classical 2D setting and found that these bacterial strains elicited different effects in terms of biochemical cell functions related to inflammation, cell metabolism, and oxidative stress. On the same line, we cannot exclude that in our setting too the use of a different SA strain might have produced different results to the ones here reported. Therefore, as a perspective it will be interesting to challenge our 3D model with other bacterial strains (even beyond the *Staphylococcus* genus), which will also allow testing specific mechanistic hypothesis. Moreover, our 3D model represents a simplified pathological scenario owing to the presence of a single skeletal cell type. While a model with reduced complexity is useful for basic understanding of biological processes, we may envisage further implementations of our model through addition of other cell types present in the bone tissue, in order to gain a broader overview of the OM pathogenesis.

## 4. Materials and Methods

### 4.1. Staphylococcus aureus Strain and Growth

The *Staphylococcus aureus* strain used in this study was an antibiotic-sensitive Newman clinical isolate [57]. SA was expanded by incubation in tryptic soy broth (TSB, Waltham, MA, USA) at 37 °C and 200 rpm, and harvested on the day of the infection experiments (see below) when an optical density at 600 nm of 0.6 was achieved (i.e., start of the exponential growth phase), corresponding to 1 × 10^8^ CFU (colony-forming unit)/mL. Optical densities were measured using a GeneQuant *pro* spectrophotometer (Amersham Biosciences, Little Chalfont, UK) and CFUs counted as detailed below. Prior to infection, bacteria were resuspended with the alpha minimum essential medium containing ribonucleosides, deoxyribonucleosides, 2 mM L-glutamine, and 1 mM sodium pyruvate (α-MEM, Waltham, MA, USA), supplemented with 10% FBS in the absence of penicillin/streptomycin (P/S) and ascorbic acid. In some experiments, SA was inactivated by overnight incubation with 2% (*w/v*) paraformaldehyde, followed by quenching with 50 mM NH_4_Cl, then washed, and resuspended with phosphate buffered saline (PBS).

### 4.2. The 2D Cultures of MC3T3-E1 Cells on Plastic Plates

The murine osteoblastic MC3T3-E1 cell line (Subclone 14) used throughout the study was a gift by Professor Fernando Gianfrancesco (CNR-IGB, Naples). Cells were expanded by incubation at 37 °C and 5% CO_2_ with α-MEM supplemented with 10% FBS and 2% penicillin/streptomycin (the culture medium was changed twice a week). For 2D cultures on plastic plates, MC3T3-E1 cells were harvested using trypsin EDTA (Sigma-Aldrich, St. Louis, MO, USA), and seeded onto uncoated 48 well cell culture plates (Corning, New York, NY, USA) at 3 × 10^5^ cells/well using the same medium without P/S.

### 4.3. The 3D Cultures of MC3T3-E1 Cells on Magnesium-Doped Hydroxyapatite/Collagen I (MgHA/Col) Scaffolds

MgHA/Col scaffolds were produced as previously described [37,38,39]. These were conditioned in 48 well cell culture plates with α-MEM without P/S for 2 h at 37 °C prior to seeding of the MC3T3-E1 cells. Conditioned scaffolds were transferred into new wells and the top surface of each was seeded with 20 μL of half the cell suspension (5 × 10^5^ cells). Plates were incubated for 15 min to allow cell attachment and scaffolds were turned over to seed the remaining cell suspension (20 μL containing 5 × 10^5^ cells) onto the opposite surface. Following a second incubation of 15 min, α-MEM without P/S was added to each well (700 μL/well) and plates were moved to the incubator. Proliferation and metabolic activity of osteoblasts were measured every 3 days up to 9 days of culturing to monitor adaptation of these cells to the MgHA/Col scaffolds. See Figure S1 in Supplementary Materials for a scheme of the experimental design.

### 4.4. Infection Experiments (SA/MC3T3-E1 2D and 3D Cocultures)

MC3T3-E1 cells, either grown on 48 well cell culture plates (2D) or on MgHA/Col scaffolds (3D), were infected with live SA at MOI (multiplicity of infection) of 160:1 (160 × 10^6^ bacteria: 1 × 10^6^ MC3T3-E1 cells). Expression of selected genes by MC3T3-E1 and SA, bacterial CFUs, concentration of ALP, OPG, TNF-α, and PTX3 in the conditioned medium and viability of the MC3T3-E1 cells were assessed at different time points up to 3 days of culturing in both 2D and 3D conditions, as detailed below. In some experiments, the metabolic activity of MC3T3-E1 cells was determined using inactivated SA.

### 4.5. Assay for Proliferation of MC3T3-E1 Cells

Proliferation of the MC3T3-E1 cells was assessed by quantifying genomic DNA using the PicoGreen assay (Invitrogen). MC3T3-E1 cells grown either on plastic plates or on MgHA/Col scaffolds were lysed using 10 mM Tris-HCl and 1 mM EDTA (TE) buffer containing 0.2% (*w/v*) Triton X-100, followed by three freeze-and-thaw cycles. Lysed samples were incubated with the PicoGreen reagent according to the manufacturer’s instructions, and fluorescence intensity was measured at 538 nm following excitation at 485 nm using a ClarioStar plate reader (BMG Labtech, Ortenberg, Germany). The contribution of bacterial DNA (DNA_SA_) to the PicoGreen signals recorded from SA/MC3T3-E1 cocultures (DNA_MC3T3-E1/SA_) was determined based on a CFU vs. DNA standard curve from monocultures of SA. MC3T3-E1 DNA (DNA_MC3T3-E1_) was then calculated by subtracting DNA_SA_ from DNA_MC3T3-E1/SA_ (i.e., DNA_MC3T3-E1_ = DNA_MC3T3-E1/SA_ − DNA_SA_).

### 4.6. Assay for Metabolic Activity of MC3T3-E1 Cells

The Alamar Blue assay (Thermo Fisher Scientific, Waltham, MA, USA) was used to measure the metabolic activity of MC3T3-E1 cells cultured in 2D or 3D conditions in the presence and absence of inactivated SA. Following addition of the Alamar Blue reagent, plates were incubated for 4 h at 37 °C and 70 rpm with 5% CO_2_. After incubation, 100 µL of conditioned medium were transferred to black 96 well plates (Corning) and fluorescence intensity was recorded at 560 nm following excitation at 590 nm using a ClarioStar plate reader.

### 4.7. CFU Counting

SA CFUs were measured both from 2D and 3D MC3T3-E1/SA cocultures. Briefly, MC3T3-E1 cells were washed three times with PBS to remove loosely adherent bacteria, then 1 mL/well of TSB was added. MC3T3-E1 cells were ruptured either by scraping (2D) or ultrasonication (3D) for 20 min at 150 W and 50 Hz in an ultrasonic bath (Ultrasonic cleaning instrument, Falc Instruments, Treviglio, Italy). Supernatants were collected, diluted 1 in 10, plated on tryptic soy agar (TSA) plates (Becton Dickinson, Milan, Italy), and incubated at 37 °C for 24 h.

### 4.8. Fluorescence Microscopy

MgHA/Col scaffolds (i.e., from 3D monocultures of either SA or MC3T3-E1 cells or 3D SA/MC3T3-E1 cocultures) were transferred to histology cassettes and kept in 4% neutral-buffered formalin for 24 h. Then they were dehydrated using increasing concentrations of alcohol (70–100% ethanol), cleared with xylene, and embedded in paraffin. The paraffin section of the scaffolds (3 µm) were deparaffinized with xylene and rehydrated using decreasing concentrations of alcohol (100–70% ethanol) and water, then washed with PBS. The samples then saturated with PBS containing 2% (*w/v*) BSA for 45 min, and incubated with a FITC-conjugated anti-*Staphylococcus aureus* antibody (Abcam, Cambridge, UK). Nuclei were stained by incubation with 30 µg/mL of propidium iodide (PI) for 5 min at room temperature (RT). Sections were then mounted onto a glass slide using Dabco mounting media, and scanned for fluorescence using a DMI8 inverted fluorescent microscope (Leica, Wetzlar, Germany).

### 4.9. Scanning Electron Microscopy (SEM)

Empty scaffolds and scaffolds coming from the monoculture and coculture experiments were harvested at different time points and washed with cold 0.1 M sodium cacodylate buffer, pH 7.4 (washing buffer). Then they were fixed with glutaraldehyde 2.5% dissolved in washing buffer and incubated for 2 h at 4 °C. After fixation, scaffolds were rinsed with washing buffer. The samples were dehydrated with passages in a series of increasing scale alcohol, followed by two final passages with hexamethyldisilazane (Sigma-Aldrich) at RT. Dehydrated samples were sputter-coated with gold (20 µm gold film) and observed by using Stereoscan 360 SEM (Cambridge Instruments, Cambridge, UK).

### 4.10. RNA Extraction

Cell-seeded MgHA/Col scaffolds were washed with PBS and placed in 350 µL of digestion buffer (0.025% trypsin, 1 mg/mL collagenase IV in HBSS) for 5 min, with gentle pipetting every 2 min. Reaction mixtures were transferred to clean wells, blocked with 350 µL of FBS + 2 µL of 1 M EDTA, and centrifuged for 5 min at 1200 rpm and RT. The resulting pellets were resuspended with 600 µL/each of Trizol (Thermo Fisher Scientific), and total RNA isolation was performed according to standard procedures. In 2D experiments, MC3T3-E1 cells were directly incubated with 250 µL/well of Trizol prior to RNA extraction.

### 4.11. Gene Expression Analysis

Reverse transcription was carried out using 1 μg of total RNA and the High Capacity cDNA Reverse Transcription Kit (Applied Biosystems, Foster City, CA, USA). Quantitative RT-PCR was performed using SsoAdvanced™ SYBR^®^ Green Supermix (Bio-Rad, Hercules, CA, USA). See Supplementary Materials for sequences of the primers used for murine (Supplementary Table S1) and bacterial (Supplementary Table S2) genes.

### 4.12. Enzyme-Linked Immunosorbent Assays (ELISA) and ALP Enzymatic Activity

The conditioned medium of both 2D and 3D SA/MC3T3-E1 cocultures was centrifuged for 5 min at 13,000 rpm and 4 °C, to remove bacteria, and the concentration of TNF-α, OPG, and PTX3 was determined using commercial ELISA kits (R&D Systems, Minneapolis, MN, USA), according to the manufacturer’s instructions. ALP enzymatic activity was quantified using the Alkaline Phosphatase Activity Colorimetric Assay kit (BioVision, Milpitas, CA, USA).

### 4.13. Statistical Analysis

Statistical analysis and graphical representation of data were carried out using GraphPad Prism version 7.0 (GraphPad Software, Inc., San Diego, CA, USA). Data are represented as mean ± standard error (SEM). Within-group comparisons were performed by one-way analysis (ANOVA) followed by Dunn’s or Sidak multiple comparisons post hoc test. Between-group comparisons were carried out using the Mann–Whitney test. Results were regarded as significantly different if the test’s two-tailed p-value was below 0.05.

## 5. Conclusions

The work presented here demonstrates that MgHA/Col matrices closely mimic the physiological environment of the natural human bone and provide a suitable scaffold for 3D models of OM. Due to their cytocompatibility and cell adhesive properties, these biomimetic scaffolds are particularly amenable to study early events in OM pathogenesis, with major regard to bacterial adhesion and OB infection. Additionally, the applied MgHA/Col matrices are intrinsically bioactive, in that they are endowed with chemical and mechanical cues that shape and inform both OB and SA biological responses. Therefore, the 3D model here developed is a close-to-in vivo representation of the host–pathogen interface in OM and stands as a novel and useful tool to study human bone infections in a relevant setting to the human pathology.

## 6. Patents

The authors declare no patents related to the objects of the study.

## Data Availability

Data supporting the reported results can be found at https://zenodo.org/communities/humanitasirccs (accessed on 1 June 2021). and will be made available upon request to the corresponding authors.

## Supplementary Materials

The following are available online at https://www.mdpi.com/article/10.3390/pathogens10070837/s1, Table S1: RT-PCR murine primers. Table S2: RT-PCR *Staphylococcus aureus* primers. Figure S1: Experimental design and outcomes of the in vitro model of OM developed in the study (MC3T3-E1/SA cocultures on MgHA/Col bone biomimetic scaffolds). Figure S2: Gene expression of SA-infected MC3T3-E1 cells in 2D cocultures.
