# Supplementary material for: 3D Cocultures of Osteoblasts and Staphylococcus aureus on Biomimetic Bone Scaffolds as a Tool to Investigate the Host–Pathogen Interface in Osteomyelitis
Source: Pathogens. 2021 Jul 3;10(7):837. doi: 10.3390/pathogens10070837 (PMC8308613; doi:10.3390/pathogens10070837)
Supplement: Supplementary file 1 [file pathogens-10-00837-s001.zip › pathogens-1264720-supplementary.pdf]

pathogens

MDPI

Article

3D Cocultures of Osteoblasts and *Staphylococcus aureus* on Biomimetic Bone Scaffolds as a Tool to Investigate the Host–Pathogen Interface in Osteomyelitis

Raffaella Parente, Valentina Possetti, Maria Lucia Schiavone, Elisabetta Campodoni, Ciro Menale, Mattia Loppini, Andrea Doni, Barbara Bottazzi, Alberto Mantovani, Monica Sandri, Anna Tampieri, Cristina Sobacchi and Antonio Inforzato

Supplementary Materials and Methods

Sequences of the primers used in the RT-PCR esperiments are listed in Table S1 (for the MC3T3-E1 murine cells) and Table S2 (for the Newmann strain of *Staphylococcus aureus* used in the study).

Table S1. RT-PCR murine primers.

Targeted Gene (mouse)	Primer Sequences (5′-3′)
18S	F: CTCAACACGGGAAACCTCAC<br>R: CGCTCCAACTAAGAAGG
Ptx3	F: CGAAATAGACAATGGACTCCATCC<br>R: CAGGCGCACGGCGT
Spp1	F: GAAGGCTCATGGTTGGATGT<br>R: GTAGCCCAAGGGTATTTCAG
Col1a1	F: CTCCTGGTATTGCTGGTGCT<br>R: TTCACCAGGAGAACCTTTGG
NRf2	F: CATGATGGACTTGGAGTTGC<br>R: CCTCCAAAGGATGTCAATCAA
Ho-1	F: AGGCTAAGACCGCCTTCCT<br>R: TGTGTTCCTCTGTCAGCATCA
Alp	F: AAGGCTTCTTCTTGCTGGTG<br>R: GGTGTATCCACCGAATGTGA
Tgf-β	F: GTCAGCAGCAGCCGGTTACCA<br>R: TGGAGCAACATGTGGAACTC
Opg	F: GTTCCCGAGGACCACAAT<br>R: CCATTCAATGATGTCCAGGAG
Tnf-α	F: GACGTGGAACTGGCAGAAGAG<br>R: TTGGTGGTTTGTGAGTGTGAG
Bmp2	F: CGGACTGCGGTCTCCTAA<br>R: GGGGAAGCAGCAACACTAGA
Zo-1	F: ATGCAGACCCAGCAAAGGT<br>R: TGACCAAGAGCTGGTTGTTTT

Table S2. RT-PCR *Staphylococcus aureus* primers.

Targeted Gene (*Staphylococcus Aureus*)	Primer Sequences (5′-3′)
16S	F: GCCACACTGGAACTGAGACA<br>R: AGTTAGCCGTGGCTTTCTGA
Spa	F: AAGAAGACGGCACGGAGTA<br>R: TTAGCATCTGCATGGTTTGC
Psm	F: TGTCATACCCCAGCAGAGTG<br>R: TAATGGCGCTTGGCTTTATT
ClfA	F: CAACTGCTAAAGTGCCACCA<br>R: GTCAATATAAGCGGGCATGG
agrRNAIII	F: AAAGTTGCAGCGATGGATTT<br>R: AAATGCGCAATGAGTCTGTG
FnbpA	F: CCAGGTGGTGGTCAGGTTAC<br>R: GTGCTTGACCATGCTCTTCA

Supplementary Figures

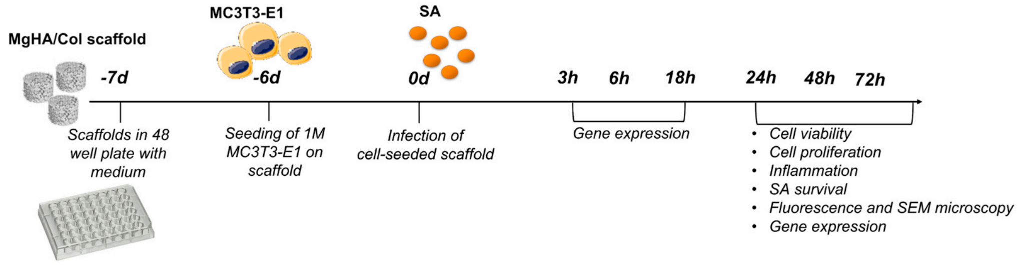

Figure S1. Experimental design and outcomes of the *in vitro* model of OM developed in the study (MC3T3-E1/SA co-cultures on MgHA/Col bone biomimetic scaffolds).

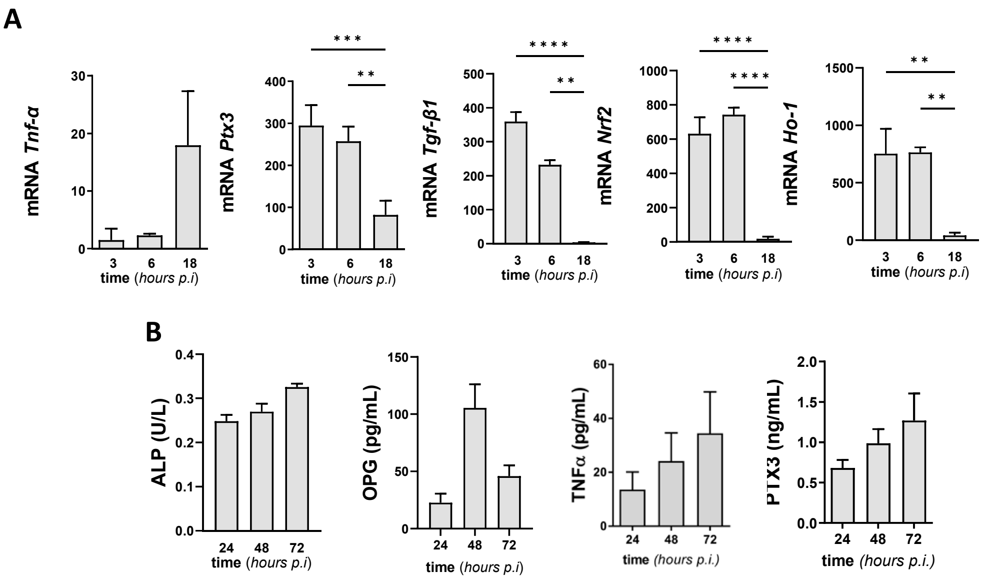

Figure S2. Gene and protein expression of SA-infected MC3T3-E1 cells in 2D co-cultures. MC3T3-E1 cells were seeded on MgHA/Col 3D scaffolds, and cultured for 6 days. Live SA was then inoculated (MOI of 160:1), and co-cultured with MC3T3-E1 cells up to 72 hours. (**A**) Cells were harvested at the reported time points, total RNA was extracted, and mRNA levels of the indicated genes were measured by qRT-PCR. Data were normalized based on the levels of 18S rRNA, and expressed as mean ± SEM (n = 4–6 from 2 to 3 independent experiments preformed in duplicate). (**B**) Activity of ALP and concentration of OPG, TNF-α, and PTX3 in the conditioned medium of MC3T3-E1/SA co-cultures were determined using commercial enzymatic and ELISA kits. Data are from 3–4 independent experiments performed in duplicate or triplicate (n = 7, mean ± SEM). In all panels, **** $p < 0.001$, *** $p < 0.005$, ** $p < 0.01$, * $p < 0.05$, one-way ANOVA test with Dunn's multiple comparisons test.
